# Immediate impact of Mindfulness-Based Cognitive Therapy (MBCT) among women with breast cancer: a systematic review and meta-analysis

**DOI:** 10.1186/s12905-023-02486-x

**Published:** 2023-06-22

**Authors:** Yun-Chen Chang, Tzuhui Angie Tseng, Gen-Min Lin, Wen-Yu Hu, Chih-Kai Wang, Yuh-Ming Chang

**Affiliations:** 1 School of Nursing and Graduate Institute of Nursing, China Medical University, 406 Taichung, Taiwan; 2 Nursing Department, China Medical University Hospital, Taichung, Taiwan; 3 Department of Environmental and Cultural Resources, National Tsing Hua University, Hsinchu, Taiwan; 4 Departments of Medicine, Hualien-Armed Forces General Hospital, Hualien City, Taiwan; 5 Tri-Service General Hospital, National Defense Medical Center, Taipei City, 100 Taiwan; 6 School of Nursing, College of Medicine, National Taiwan University, 100, Taipei City, Taiwan; 7 Department of Nursing, National Taiwan University Hospital, Taipei City, 100 Taiwan; 8 Cancer Center, China Medical University Hospital, Taichung, 404 Taiwan; 9 Department of Neurology, Hsinchu Mackay Memorial Hospital, No. 690, Sec. 2, Guangfu Rd., East Dist., Hsinchu City, 30071 Taiwan

**Keywords:** Mindfulness-based cognitive behavioral therapy, MBCT, Oncology, Systematic review, Meta-analysis

## Abstract

**Background:**

Mindfulness-based cognitive therapy (MBCT) may have positive physiological and psychological benefits for breast cancer survivors. However, few studies involved a combination of the relevant literatures to confirm the effects.

**Methods:**

Our study included randomized controlled trials (RCTs) and non-RCTs comparing interventions of MBCT and control protocols for alleviation of symptoms among breast cancer survivors. We calculated pooled mean differences (MDs), standardized mean differences (SMDs), and 95% confidence intervals (CIs) by using random effects models to estimate summary effect sizes.

**Results:**

Thirteen trials with 20–245 participants were considered in our studies; for the meta-analysis, 11 of these studies were eligible for assessment. The pooled meta-analysis results revealed that at the end of the MBCT intervention, participants’ anxiety (SMD, − 0.70; 95% CI, − 1.26 to − 0.13; *I*^2^ = 69%), pain (SMD, − 0.64; 95% CI, − 0.92 to − 0.37; *I*^2^ = 0%), and depression (SMD, − 0.65; 95% CI, − 1.14 to − 0.17; *I*^2^ = 75%) levels significantly decreased, and their mindfulness (MD, 8.83; 95% CI, 3.88 to 13.78; *I*^2^ = 68%) levels significantly increased.

**Conclusion:**

The MBCT may be associated with improved pain, anxiety, depression, and mindfulness. However, the quantitative analysis pointed to an inconclusive result due to moderate to high levels of heterogeneity among indicator of anxiety, depression, and mindfulness. Future work requires more studies to better elucidate the clinical significance of this possible association. The results suggest that MBCT is highly beneficial as an intervention for patients who have received treatment for breast cancer.

**Supplementary Information:**

The online version contains supplementary material available at 10.1186/s12905-023-02486-x.

## Background

According to a 2020 Global Cancer Statistics report, breast cancer is the commonest cancer worldwide and the main cause of cancer-related mortality in women [1]. As per Taiwan’s Ministry of Health and Welfare, in Taiwan, cancer of the breast is the commonest type of cancer in women [2]. Because of the use of new cancer treatments over the past decades, the life expectancy of individuals with breast cancer has increased. However, those who survive breast cancer commonly develop psychosocial and physical complications, such as sleep disturbance, fatigue, pain, and psychological distress [3–8]. In breast cancer survivors, the aforementioned complications may negatively affect their overall health-related quality of life (QOL) and may influence treatment outcomes. A study demonstrated that a symptom cluster of anxiety, depression, fatigue, and pain adversely affected the QOL of women with breast cancer who were receiving radiotherapy or chemotherapy [9]. Evidence suggests that breast cancer survivors at any stage often experience fear of cancer recurrence (FCR), which may negatively affect their QOL [10, 11]. Another study revealed that breast cancer survivors with depressive symptoms tend to have a lower treatment adherence than those without such symptoms [12]. A meta-analysis revealed that depression in breast cancer survivors has a significant association with cancer recurrence, cancer-specific mortality, and all-cause mortality; moreover, anxiety in breast cancer survivors is associated with cancer recurrence and all-cause mortality but not with cancer-specific mortality [13].

Practitioners have increasingly applied mindfulness-based interventions (MBIs) in clinical settings to reduce the negative psychological effects of cancer and its treatment [14]. Mindfulness-based cognitive therapy (MBCT) and mindfulness-based stress reduction (MBSR) are the most common structured MBIs. MBCT integrates mindfulness practice with elements of cognitive behavioral therapy that distinguishes MBCT from other MBIs [15, 16]. In recent years, several clinical studies examined the effect of MBCT in patients with breast cancer [17–20]. The findings of these studies on MBCT are inconsistent. For example, Johannsen et al. reported MBCT significantly reduced pain and had an effect on QOL, but found no statistically significant effects on psychological distress [17]. In another study, Park et al. reported MBCT had a significant effect on psychological distress (anxiety and depression), FCR, fatigue, spiritual well-being, and QOL [18]. Previous systematic reviews and meta-analyses had revealed that MBIs represent effective treatment options for women with breast cancer [21–23]. However, the majority of studies evaluated the effects of both MBCT and MBSR rather than the effects of MBCT. Thus, the effectiveness of MBCT in female patients with breast cancer remains unconfirmed. In our study, we performed a systematic meta-analysis of available evidence related to the treatment effects of MBCT in female patients with breast cancer.

### Research question

Our study examined the following research question: What are the effects of MBCT on psychological, physiological, QOL, and clinical outcomes among patients with breast cancer?

## Materials and methods

We registered the present review on the International Prospective Register of Systematic Reviews (PROSPERO; registration number = CRD42022301045).

### Database and search strategy

The review procedures, including its design, adhered to the Preferred Reporting Items for Systematic Reviews and Meta-Analyses guidelines [24]. The two authors searched for all studies published before December 2021 in various databases, including Embase, PubMed, PubMed Central (PMC), CINAHL, and PsycINFO. Randomized controlled trails (RCTs) and non-RCTs (e.g., single-group, quasi-experimental research design), and the following search terms were employed: “breast,” “breast cancer,” “breast neoplasms,” “MBCT,” “mindfulness-based cognitive therapy,” “clinical trials,” and “within 10 years.” Identified title and abstracts were screened independently by two authors (YC and YM). Any disagreements were settled through discussion with a third author (TA) until consensus was achieved.

### Exclusion and inclusion criteria

The PICOS (participants, interventions, comparators, outcomes, and study type) framework was employed to establish the inclusion criteria for both RCT and non-RCT studies, without any language restrictions, to encompass all relevant studies. Women with breast cancer who had undergone MBCT (with a threshold of at least 60% of breast cancer patients in the literature, if the original recruitment included more cancer types) were included in the study, and patients in the control group (or without control group) who had not received MBCT were included for comparison. The results of interest were the physical and psychological statuses of patients with breast cancer after Western medicine–based drug treatment (e.g., sleep quality, QOL, depression, anxiety, FCR, pain, mindfulness, stress, fatigue, and sexual function). We excluded conference abstracts, observational studies, studies not involving human participants, and protocol studies.

### Extraction of data and assessment of data quality

The aforementioned 2 authors independently extracted and coded the data from the studies included in this review. The following data were collected: publication year, first author’s name, country in which research was conducted, patient diagnosis, and number of participants. The authors also collected data on the MBCT protocol, namely presence or absence of a control group, outcome variables, follow-up duration, and study results. Only data published in relevant articles were considered suitable for data extraction. We obtained mean and standard deviation values after the MBCT intervention and control group protocol, and the sample size of both groups was considered in our analysis. We used the Cochrane risk-of-bias tool to examine the RCTs included in this study [25].

### Statistical analysis

RevMan (version 5.4.1; Cochrane Community, London, UK) was used for statistical analyses [26]. At the post-intervention time-point, data were pooled. When different rating instruments were used in studies, we employed the standardized mean difference (SMD) to reveal the study effect size; when the same rating instruments were used, we employed mean differences (MDs) and 95% confidence intervals (CIs) for continuous outcome summaries. Because the trails we included were clinically and statistically heterogeneous, we assumed that the effect size was different; therefore, for outcome measurement, the random effects model of DerSimonian and Laird [27] was employed. We examined heterogeneity by using the Cochrane Q test and* I*^2^; the *I*^2^ value range was 0%–100%. *I*^2^ values of 75%, 50%, and 25%, respectively, indicated high, moderate, and low heterogeneity. Statistical significance was indicated by a P value below 0.05.

## Results

### Study descriptions and quality assessments

**Fig. 1:**
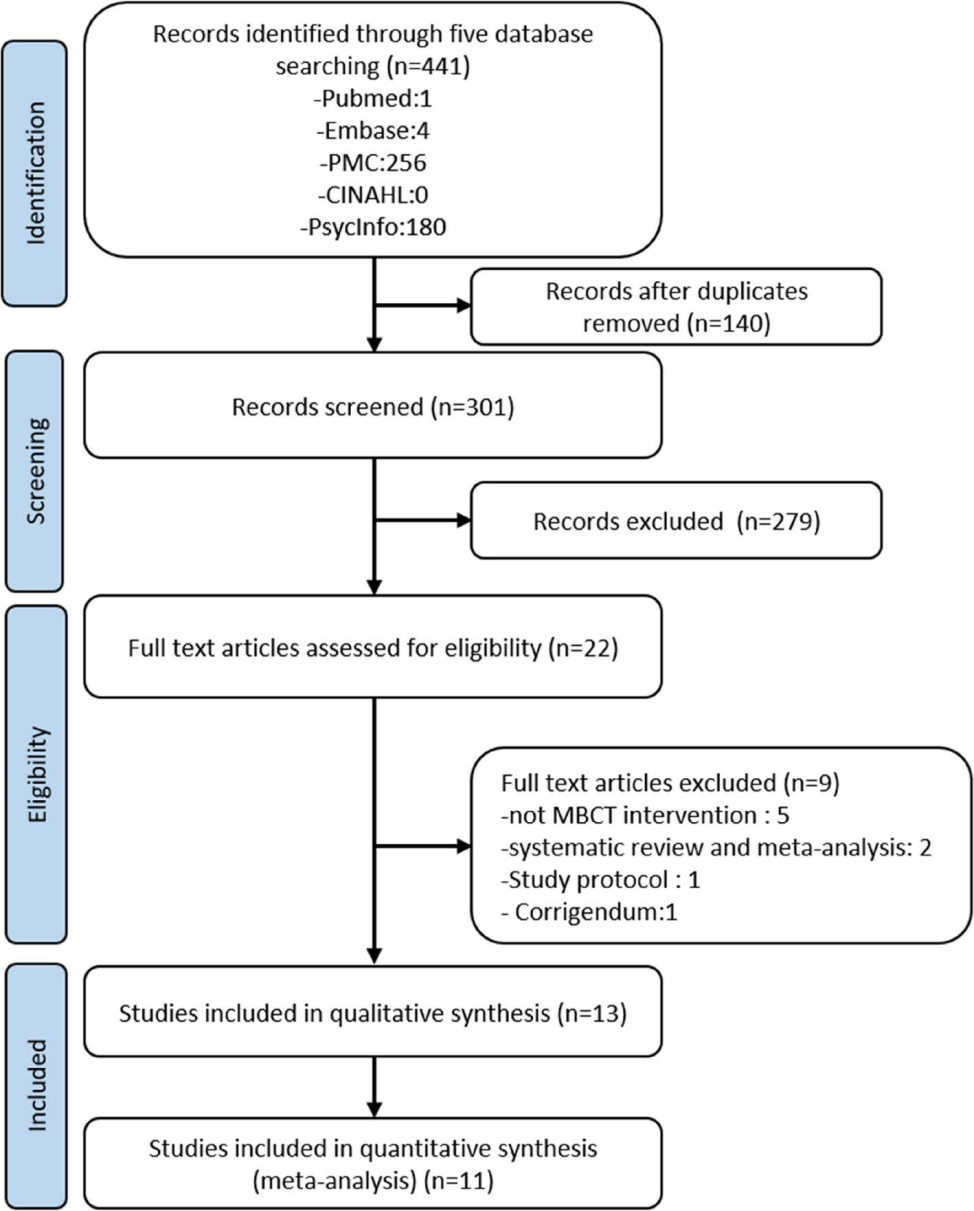
The process of literature search

In total, 441 articles were systematically searched via Pubmed, Embase, PMC, CINAHL, and PsycInfo. After removing 140 duplicate entries, the remaining 301 articles were screened for their abstracts, content and titles. Out of the total of 301 articles reviewed, it was found that 279 articles did not meet the inclusion criteria, as they did not recruit patients with breast cancer or employ the MBCT intervention. Consequently, these articles were further excluded from the analysis. We deemed 22 studies to be eligible for a complete screening. After reading the full text of 22 articles, a total of 9 articles were excluded as they pertained to a MBCT intervention (*n* = 5), systematic review and meta-analysis (*n* = 2), study protocol (*n* = 1), and corrigendum (*n* = 1). Finally, 13 studies (10 RCTs and 3 non-RCTs) satisfied our inclusion criteria and were subjected to a qualitative synthesis. Of those studies, 11 had complete data, and we included them in our meta-analysis. Figure 1 described the search algorithm [28].

### Methodological quality of studies

**Fig. 2:**
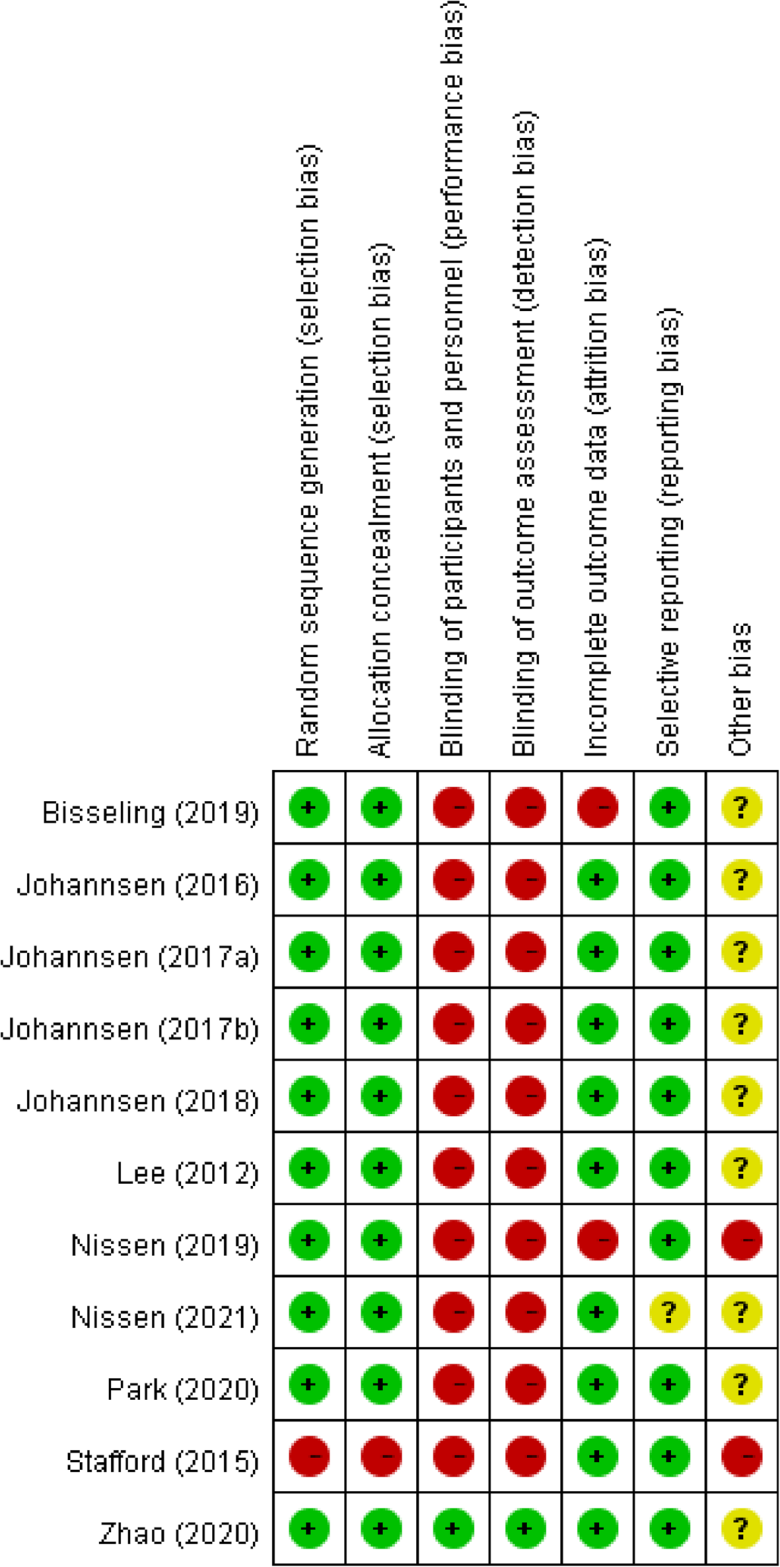
Cochrane risk‐of‐bias summary for included studies

Figure 2 presented a summary of our quality assessments. We indexed studies according to their year of publication and the first author’s surname. We considered 7 risk-of-bias domains for each study. In the domain of random sequence generation, 90.9% (10/11) of studies exhibited a low risk of selection bias. In the allocation concealment domain, 10 out of 11 studies (90.9%) exhibited a low risk of selection bias; only one study had a high risk of selection bias. In the domain of participant and personnel blinding, 1 study exhibited a low risk of performance bias, with the other 10 exhibiting a high risk. Regarding outcome assessment, 90.9% (10/11) of studies were revealed to have a high risk of detection bias. For the incomplete outcome data domain, a low risk of attrition bias was noted for 81.8% (9/11) of studies. For selective reporting, 90.9% (10/11) of studies had a low risk of reporting bias. In the “other bias” domain, 81.8% (9/11) of studies exhibited an unclear bias risk.

### Characteristics of eligible studies

The basic features of 13 trials (10 RCTs and 3 non-RCTs) are presented in Table 1. The trials took place in Denmark (6 trials), the Netherlands (2 trials), Australia (2 trials), Japan (1 trial), China (1 trial), and the United States (1 trial). Eight trials for cancer patients were in the treatment phase to complete treatment, 4 trials recruited current cancer treatment or active follow-up, and 1 trial did not specify treatment phase. At least 60% of patients diagnosed with breast cancer (six studies additionally included non-breast cancer type), and the sample size range was 20–245. The main form of teaching was face-to-face group tutoring (11 trials) [17–20, 29–34]. Participants in 2 studies participated in individualized MBCT sessions led by experienced online therapists [35, 36].

**Table 1:** Characteristics of trials included in the systematic review

Author/ year/ Country	Participants	Phase of treatment	Design	Intervention	protocol	Control group	Follow up	Outcome variables	Results
Nissen (2021) Denmark[36]	*N* = 82 Breast (*n* = 75,91.5%) and prostate cancer (*n* = 7,8.5%)	Completed primary treatment	RCT	individual internet‐delivered MBCT	9 weeks, to complete the 8 modules	wait‐list control	baseline, 5 weeks, 10 weeks (post intervention), and 6 months	BDI,, STAI‐Y, Demographic predictors, WAI-C, FFMQ-SF, SCS-SF	1.higher baseline depression level was associated with an increased response to treatment for anxiety after treatment 2.lower level of self-compassion was associated with an increased response to treatment for depression after treatment
Park (2020) Japan [18]	*N* = 74 Breast cancer	NA	RCT	face‐to‐face group MBCT	8 week × 2 h/wk.; Homework 20–45 min	wait‐list control	baseline (T0), Week 8 (T1), and Week 12 (T2)	HADS, CARS, BFI, FACIT-Sp, FACT-G, FFMQ	**Significant:** psychological distress, FCR, fatigue spiritual well-being, QOL The beneficial effect persisted for up to four weeks following the completion of the intervention
Zhao (2020) China [29]	*N* = 136 Breast cancer	completed therapy ≥ 1 month before study enrolment	RCT	Group-based MBCT	*8–10 participants over 6 weekly 90-min sessions, for a total of nine contact hours*	wait‐list control	baseline (T1), post-intervention (T2), 3-month (T3) and 6-months (T4)	ISI, FFMQ	**Significant:** Improved insomnia, as well as increased total sleep time, sleep efficiency and mindfulness; decreased sleep onset latency and waking after sleep onset
Bober (2020) USA [34]	*N* = 20 young breast cancer/35.6 (SD = 6.49)	currently receiving medication to induce OS	One-group interventional study	MBCT + sexual health rehabilitation, body awareness exercises	single 4-h group intervention	NA	baseline and 2 months	FSFI, BSI-18, GSI	**Significant:** Pre- and post-intervention analyses showed significant improvements in female sexual health and anxiety at 2 months compared to baseline Moderate-to-large effect sizes were observed for sexual function and psychological distress changes
Nissen (2019) Denmark [35]	*N* = 150 Breast (*n* = 137,91.3%) and others cancer(n = 13,8.7%)	completed primary treatment	RCT	individual Internet‐delivered MBCT	eight 1 week modules	wait‐list control	baseline, 5 weeks, 10 weeks (post intervention), and 6 months	BDI, ISI, PSS, STAI‐Y, WHO‐5	**Significant:** anxiety, depressive symptoms Follow-up assessments indicated sustained effects on anxiety, but not on depressive symptoms
Bisseling (2019) Netherland [30]	*N* = 120 Breast(*n* = 75,62.5%) and others cancer(n = 45,37.5%)	currently receiving treatment	Part of a larger multicentre RCT	face‐to‐face group MBCT	8 week × 2.5‐hr, one 6‐hr silent day	usual (TAU)	Baseline, 8 weeks	HADS, GCQ‐22, WAI	**Significant:** 1.psychological distress decreased 2.therapeutic alliance did predict reduction of psychological distress
Johannsen (2018) Denmark [31]	*N* = 129 Breast cancer	completed treatment	RCT	Group-based MBCT	*8-week*	wait‐list control	baseline(T1) postintervention after the 8-week (T2), 3 (T3) and 6 months (T4)	FFMQ, SCS-SF, PCS	**Significant:** indirect effects were found for mindfulness nonreactivity and pain catastrophizing **No Significant:** indirect effect was found for self-compassion
Johannsen (2017a) Denmark [19]	*N* = 129 Breast cancer	completed treatment	RCT	Group-based MBCT	8 week × 2 h/wk	wait‐list control	baseline(T1) postintervention after the 8-week (T2), 3 (T3) and 6 months (T4)	Costs (€), MCID	MBCT cost was 240€ per participant which was a cost‐effective pain intervention
Johannsen (2017b) Denmark [19]	*N* = 129 Breast cancer	completed treatment	RCT	Group-based MBCT	*8-week*	wait‐list control	baseline(T1) postintervention after the 8-week (T2), 3 (T3) and 6 months (T4)	HADS, ECR-SF, TAS-20	**Significant:** attachment avoidance as a moderator- Compared with a lower level of attachment avoidance, a higher level of attachment avoidance predicts that MBCT is more effective in reducing ***pain intensity***
Johannsen (2016) Denmark [17]	*N* = 129 Breast cancer	completed treatment	RCT	Group-based MBCT	8 week × 2 h/wk	wait‐list control	baseline(T1) postintervention after the 8-week (T2), 3 (T3) and 6 months (T4)	HADS, SF-MPQ-2, PPI, NRS, WHO-5	**Significant:** MBCT reduced pain intensity, neuropathic pain, and the PPI **No Significant:** affective pain dimensions or the two sensory pain dimensions of continuous and intermittent pain
Stafford (2015) Australia [20] **(pilot study)**	*N* = 66 Breast (*n* = 45, 68.2%) and gynecologic cancer (*n* = 21,31.8%)	current cancer treatment or active follow-up	Quasi-experimental research design	Group-based MBCT	8 week × 2 h/wk	MMP	Baseline, postintervention after the 8-week, 3 months	DASS-21, FACT-G, FMI	**Significant:** improved distress, QOL, and mindfulness in MBCT and MMP intervention
Stafford (2013) Australia [32]	*N* = 42 Breast (*n* = 30,71%) and gynecologic cancer(*n* = 12,29%)	current cancer treatment	One-group interventional study	Group-based MBCT	8 week × 2 h/wk	NA	Baseline, postintervention after the 8-week, 3 months	DASS-21, FACT-G, FMI, PTGI	**Significant:** improved with large effect sizes in distress, QOL, mindfulness and post-traumatic growth, and maintained for 3 months
Van der Lee (2012) Netherlands [33]	*N* = 83 Breast (*n* = 50, 60%) and others cancer(*n* = 33,40%)	completed treatment	RCT	Group-based MBCT	8 week × 2.5‐hr, one 6‐hr silent day + 2.5 h follow-up session 2 months after the ninth session	wait‐list control	Baseline, postintervention after the 9-week, 6 months	CIS, SIP, HDI	**Significant:** significantly lower fatigue scores and higher well-being scores at post-treatment compared to the waiting list group. The treatment effect was sustained at the 6-month follow-up **No Significant:** found between the two groups in terms of functional impairment

*BDI* Beck depression inventory, *BFI* Brief fatigue inventory, *BSI-18* Brief symptom inventory-18, *CARS* Concerns about recurrence scale, *CIS* Checklist individual strength, *DASS-21* Depression, anxiety, stress scale, *ECR-SF* Experiences in close relationships short form, *FACT-G* Functional assessment of cancer therapy-general, *FACIT-Sp* Functional assessment of chronic illness therapy–spiritual, *FSFI* Female sexual function index, *FFMQ-SF* five facet mindfulness questionnaire-short form, *FMI* Freiburg mindfulness inventory, *GCQ‐22* Group cohesion questionnaire, *GSI* Global severity index, *HDI* health and disease-inventory, *HADS* Hospital anxiety and depression scale, *ISI* Insomnia severity index, *MBCT* Mindfulness based cognitive therapy, *MMP* Mindfulness meditation program, *MCID* Minimal clinically important difference, *MPQ* McGill pain questionnaire, *NRS* Numerical rating scale, *NA* Not applicable, *OS* Ovarian suppression, *PPI* Present pain intensity, *PCS* Pain catastrophizing scale, *PSS* perceived stress Scale, *PTGI* Posttraumatic growth inventory, *RCT* Randomized controlled trial, *SCS-SF* Self-compassion scale-short-form, *STAI‐Y* State‐trait anxiety inventory Y‐form, *SIP* Sickness impact profile, *TAS-20* Toronto alexithymia scale, *WAI* Working alliance inventory, *WAI-C* working alliance inventory – client form, *WHO‐5* World health organization 5‐item well‐being index

### Clinical trial protocol and follow-up interval

According to the data in Table 1, the MBCT programs usually lasted 8 weeks; each weekly session lasted 2 to 2.5 h and had a group-based format [17–20, 30, 32, 33]. Generally, 4 measurement time-points were employed, including baseline and post-intervention measurements. Patients were followed-up for 2 to 6 months after the intervention. In recent years, researchers have used Internet‐delivered MBCT interventions [35, 36] to replace face-to-face group meetings. In one study, an Internet‐delivered MBCT program provided an optional 1-week break, which gave participants 9 weeks to complete 8 therapist-guided sessions [36].

### Publication bias

Because relatively few studies were included in our meta-analysis, we did not conduct a funnel plot–based test of publication bias [37].

### Data synthesis and meta-analysis

#### Anxiety

**Fig. 3:**
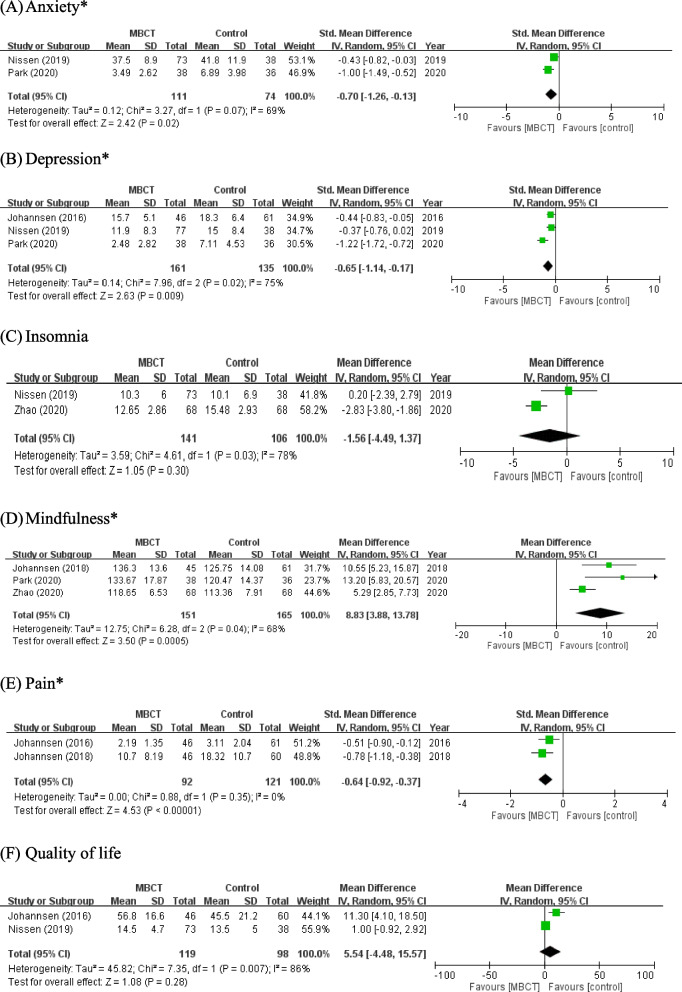
Forest plot of effect of MBCT outcome. CI, confidence interval; Std, Standardized mean difference; IV, interval variable; MBCT, Mindfulness-Based Cognitive Therapy; *Statistically significant effect (*P* < .05)

Two RCTs [18, 35] compared the immediate effects of MBCT on the anxiety of 185 participants in total. Substantial heterogeneity was identified among the studies included (*P* = 0.07,* I*^2^ = 69%). Therefore, a random effects model was applied to account for heterogeneity among study results. The MBCT group displayed significantly lower levels of anxiety (SMD =  − 0.70; 95% CI, − 1.26 to − 0.13; *P* = 0.02) than did the control cohort [Fig. 3 (A)].

#### Depression

Three RCTs [17, 18, 35] in which a total of 296 patients were enrolled reported that MBCT had immediate effects on depression symptoms. The study results were heterogeneous (*P* = 0.02, *I*^2^ = 75%); hence, a random effects model was applied to account for heterogeneity among study results. The pooled SMD was − 0.65 (95% CI, − 1.14 to − 0.17, *P* = 0.009), indicating that MBCT conferred a statistically significant effect on patients with depression symptoms [Fig. 3 (B)].

#### Insomnia

Two RCTs [29, 35] (n = 247) reported that MBCT had immediate effects on insomnia. The study results were heterogeneous (*P* = 0.03, *I*^2^ = 78%), and for this reason, a random effects model was employed. In comparison with the control group, insomnia was nonsignificantly lower in the MBCT group (pooled MD =  − 1.56; 95% CI, − 4.49 to 1.37; *P* = 0.30) [Fig. 3 (C)].

#### Mindfulness

We pooled 3 RCTs (*n* = 316) for a meta-analysis to investigate the immediate impact of MBCT on mindfulness. Heterogeneous results (*P* = 0.04, *I*^2^ = 68%) were obtained, leading us to employ a random effects model. The MBCT group exhibited significantly increased mindfulness (MD = 8.83; 95% CI, 3.88 to 13.78; *P* < 0.001) in comparison with the control group [Fig. 3 (D)].

#### Pain

Two RCTs [17, 31] (*n* = 213) were used to investigate the immediate effect of MBCT on pain. Heterogeneity was not identified between the studies included (*P* = 0.35, *I*^2^ = 0%). We applied a random effects model for estimations of heterogeneity among study results. The pooled results indicated that the MBCT intervention alleviated pain to a greater extent than did the control protocols (SMD =  − 0.64, 95% CI, − 0.92 to − 0.37; *P* < 0.001) [Fig. 3 (E)].

#### Quality of life, QOL

Two RCTs [17, 35] (*n* = 217) were applied to assess the instant effects of MBCT on QOL. In terms of short-term effects, the results exhibited heterogeneity (*P* = 0.007, *I*^2^ = 86%). Therefore, we applied a random effects model. According to our pooled results, MBCT groups did not exhibit a significant short-term improvement in QOL compared with a control protocol (SMD = 5.54, 95% CI, − 4.48 to 15.57, *P* = 0.28) [Fig. 3 (F)].

## Discussion

One strength of our meta-analysis was that in terms of immediate outcomes, it revealed that MBCT helped to reduce anxiety, depression, and pain and increase mindfulness. We applied a comprehensive search of 5 databases without the imposition of language restrictions. Furthermore, appropriate statistical analysis approaches were used for examining studies that used the same or different scales.

Anxiety and depression were prevalent comorbidities among breast cancer patients, and their presence was associated with increased mortality and cancer recurrence [13]. Our study's results were consistent with previous meta-analyses that demonstrated the beneficial effects of mindfulness-based cognitive therapy (MBCT) on major depression and anxiety symptoms when compared to control conditions [35, 38]. However, the pooled outcome of anxiety and depression showed medium heterogeneity. Possible reasons for this could include differences in the duration, mode, and type of intervention provider. On the other hand, our findings suggest the need for additional studies to directly compare the effects of mindfulness-based cognitive therapy (MBCT) and cognitive-behavioral therapy (CBT) on anxiety and depression in patients with breast cancer. Such research could have provided a more comprehensive understanding of the optimal psychosocial interventions for addressing these common comorbidities in this patient population [39, 40]. Up to 60% of patients with breast cancer experienced sustained acute pain after surgery [41]. In a previous meta-analysis, we examined five studies involving MBSR to determine whether it alleviated the pain of patients with breast cancer [42]; no significant improvements were observed. The current study demonstrated that MBCT played a significant role in reducing cancer-induced pain. However, only two articles were pooled without heterogeneity [17, 31]. Preliminary research suggested that MBCT might be a more effective intervention than MBSR for reducing pain in women undergoing breast cancer treatment. However, more evidence was needed to confirm this.

Mindfulness can support the cultivation of a compassionate attitude and foster awareness of is the state of one's mind. It may help patients with cancer gain insight into the meaning of life [18]. Three studies involving 316 participants with a diagnosis of breast cancer revealed that MBCT interventions significantly increased patients’ mindfulness [18, 29]. Unlike in previous systematic reviews or meta-analysis focusing on mindfulness, the present study considered indicators of mindfulness. Therefore, the present study produced novel findings [21–23].

Different from previous studies, we did not demonstrate that MBCT had significant effects on insomnia and QOL [18, 29]. A possible explanation could be that the two studies we included both used the Insomnia Severity Index (ISI) scale, which had a maximum and minimum of 0 and 28 points, respectively. A higher score indicated more severe insomnia [43]. However, the post-intervention scores indicated mild insomnia, which suggested a possible floor effect. Solutions for overcoming this effect could include including patients most likely to benefit from treatment, providing a more comprehensive screening program, or applying a higher cut-off point for patient screening. Further evidence is required to elucidate the mechanisms and conditions that help maintain the effects of such interventions. A possible explanation for QOL being nonsignificantly affected could be the short-term nature of the MBCT intervention; perhaps a long-term (at least 3 months) MBCT intervention is required for significant improvements. Johannsen et al. (2016) indicated that, at 3 and 6 months, the MBCT intervention groups had reported QOL-related improvements of 10.8% and 9.7%, respectively [17]. These results indicated that the improvements in QOL had been clinically significant [17].

Among the 13 included studies, in 11 of them, the MBCT intervention was in a group setting; in 2 studies, Internet-based one-on-one sessions were held. Group-based settings of mindfulness-based interventions were beneficial to patients with cancer [44, 45], and peer support facilitated the learning process [46]. The advantage of using one-on-one internet-delivered MBCT training was that numerous participants could be enrolled (thus increasing the sample size) [35], low cost [47], suitability for people with low sensory awareness [48], and the ability of participants to allocate time to practice techniques. Internet-based one-to-one MBCT may be particularly valuable for aging populations. Because older age is a significant predictor of learning loss; however, older adults may be unable to return to the questionnaire, lack motivation, and lack sufficient IT skills to complete the intervention. One study showed that cancer patients preferred face-to-face MBSR intervention in a group setting [47].

Many young patients with breast cancer faced the challenging decision of undergoing ovarian suppression and abrupt premature menopause to lower the risk of cancer recurrence [34]. Few studies have examined sexual function indicators among women with breast cancer. Such indicators were only used in one article examined in our study; thus, pooled data were unavailable. One study in our qualitative synthesis involved an integrative intervention (MBCT, sexual health rehabilitation, body awareness exercises), and the results revealed that female sexual health improved significantly after such an intervention [34]. A major concern with the aforementioned study is that the integrative intervention training rendered it difficult to determine the extent to which noted improvements could be attributed to MBCT.

Our study provides evidence that MBCT has benefits for patients with breast cancer in terms of alleviating anxiety, pain, and depression and improving mindfulness; we have provided the best available evidence on the efficacy of MBCT for alleviating various symptoms in those with breast cancer.

### Clinical implications

MBCT is a universal intervention for women undergoing breast cancer treatment or who have recently completed cancer treatment [19]. While our study found no reported adverse effects of MBCT in the 13 trials analyzed, caution should be exercised when considering its use as an additional intervention for individuals with breast cancer due to the high risk of performance and detection bias in many of the studies. Therefore, the effectiveness of MBCT as a treatment option for this population remains unclear. Future research should explore the potential benefits of MBCT on self-compassion, intimacy, and treatment alliances in breast cancer survivors. These investigations could help to establish the effectiveness of MBCT as a treatment option for this population.

### Strength and limitations

A strength of our study is that 5 databases were consulted for our comprehensive systematic review and meta-analyses. We revealed that MBCT can alleviate anxiety, depression, and pain and increase mindfulness. Substantial cultural differences existed between the participants involved in the included studies, which may have been the reason for the high degree of heterogeneity in the study results; because of the significant improvements in anxiety, depression, and mindfulness among participants, this culture-related heterogeneity can be overlooked. However, our systematic review and meta-analysis has several limitations. First, the review only included published studies, which may have been affected by publication bias, thereby limiting the generalizability of our findings. Second, for some studies, we did not have access to raw data, and consequently, we had to exclude them from our meta-analysis. This limitation restricted the number of studies included in our analysis and may have affected the reliability of our results. Third, our analysis only focused on immediate effects, and we did not examine the long-term effects of MBCT interventions. Future studies should collect relevant long-term data on the effectiveness of MBCT in reducing anxiety, insomnia, pain, and improving the quality of life in breast cancer survivors. Finally, the high risk of performance and detection bias in many of the studies analyzed may have influenced the results of our review and should be taken into consideration when interpreting our findings.

## Conclusions

The systematic review and meta-analysis conducted in this paper revealed that MBCT interventions can significantly reduce anxiety, depression, and stress symptoms among women with breast cancer. Additionally, the study found that MBCT can improve mindfulness and alleviate pain. Based on these findings, the authors suggest that MBCT could be a valuable complementary therapy for women with breast cancer, especially during the early stages of diagnosis and treatment.

## Supplementary Information

**Additional file 1.**

## Data Availability

The datasets used and/or analyzed during the current study are available from the first author (YCC, e-mail:lisacow@mail.cmu.edu.tw) on reasonable request.

## Abbreviations

BDI: Beck depression inventory
BFI: Brief fatigue inventory
BSI-18: Brief symptom inventory-18
CARS: Concerns about recurrence scale
CIS: Checklist individual strength
DASS-21: Depression, anxiety, stress scale
ECR-SF: Experiences in close relationships short form
FACT-G: Functional assessment of cancer therapy-general
FACIT-Sp: Functional assessment of chronic illness therapy–spiritual
FSFI: Female sexual function index
FFMQ-SF: Five facet mindfulness questionnaire-short form
FMI: Freiburg mindfulness inventory
GCQ‐22: Group cohesion questionnaire
GSI: Global severity index
HDI: Health and disease-inventory
HADS: Hospital anxiety and depression scale
ISI: Insomnia severity index
MBCT: Mindfulness based cognitive therapy
MMP: Mindfulness meditation program
MCID: Minimal clinically important difference
MPQ: McGill pain questionnaire
NRS: Numerical rating scale
NA: Not applicable
OS: Ovarian suppression
PPI: Present pain intensity
PCS: Pain catastrophizing scale
PSS: Perceived stress scale
PTGI: Posttraumatic growth inventory
RCT: Randomized controlled trial
SCS-SF: Self-compassion scale-short-form
STAI‐Y: State‐trait anxiety inventory Y‐form
SIP: Sickness impact profile
TAS-20: Toronto alexithymia scale
WAI: Working alliance inventory
WAI-C: Working alliance inventory – client form
WHO‐5: World health organization 5‐item well‐being index
CI: Confidence interval
Std: Standardized mean difference
IV: Interval variable
MBCT: Mindfulness-based cognitive therapy
