# Supplementary material for: Immediate impact of Mindfulness-Based Cognitive Therapy (MBCT) among women with breast cancer: a systematic review and meta-analysis
Source: BMC Womens Health. 2023 Jun 22;23:331. doi: 10.1186/s12905-023-02486-x (PMC10288664; doi:10.1186/s12905-023-02486-x)
Supplement: Supplementary file 1 — Additional file 1. [file 12905_2023_2486_MOESM1_ESM.docx]

**Search Strategy**

EMBASE

| Search number | Query | Results |
| --- | --- | --- |
| 3 | ('breast,' OR 'breast cancer,' OR 'breast neoplasms,') AND ('mbct,' OR 'mindfulness-based cognitive therapy,') AND [clinical trial]/lim AND [within 10 years]/py | 4 |
| 2 | 'mbct,' OR 'mindfulness-based cognitive therapy,' | 1284 |
| 1 | 'breast,' OR 'breast cancer,' OR 'breast neoplasms,' | 964147 |

PubMed

| Search number | Query | Results | Time |
| --- | --- | --- | --- |
| 5 | ((((MBCT) OR (mindfulness-based cognitive therapy) AND ((y_5[Filter]) AND (randomizedcontrolledtrial[Filter] OR systematicreview[Filter]))) AND (((breast) OR (breast cancer)) OR (breast neoplasms) AND ((y_5[Filter]) AND (randomizedcontrolledtrial[Filter] OR systematicreview[Filter]))) AND ((y_5[Filter]) AND (randomizedcontrolledtrial[Filter] OR systematicreview[Filter]))) AND (clinical trials) AND ((y_5[Filter]) AND (randomizedcontrolledtrial[Filter] OR systematicreview[Filter]))) AND (within 10 years) | 1 | 09:19:53 |
| 4 | (((MBCT) OR (mindfulness-based cognitive therapy) AND ((y_5[Filter]) AND (randomizedcontrolledtrial[Filter] OR systematicreview[Filter]))) AND (((breast) OR (breast cancer)) OR (breast neoplasms) AND ((y_5[Filter]) AND (randomizedcontrolledtrial[Filter] OR systematicreview[Filter]))) AND ((y_5[Filter]) AND (randomizedcontrolledtrial[Filter] OR systematicreview[Filter]))) AND (clinical trials) | 33 | 09:19:13 |
| 3 | ((MBCT) OR (mindfulness-based cognitive therapy) AND ((y_5[Filter]) AND (randomizedcontrolledtrial[Filter] OR systematicreview[Filter]))) AND (((breast) OR (breast cancer)) OR (breast neoplasms) AND ((y_5[Filter]) AND (randomizedcontrolledtrial[Filter] OR systematicreview[Filter]))) | 43 | 09:17:44 |
| 2 | (MBCT) OR (mindfulness-based cognitive therapy) | 779 | 09:15:42 |
| 1 | ((breast) OR (breast cancer)) OR (breast neoplasms) | 7,810 | 09:14:48 |

PubMed Central (PMC)

| Search | Query | Items found | Time |
| --- | --- | --- | --- |
| #4 | Search (((((breast) OR breast cancer) OR breast neoplasms)) AND ((MBCT) AND mindfulness-based cognitive therapy)) AND ((clinical trials) AND within 10 years) | 256 | 10:13:01 |
| #3 | Search (clinical trials) AND within 10 years | 880387 | 10:12:37 |
| #2 | Search (MBCT) AND mindfulness-based cognitive therapy | 1988 | 10:11:58 |
| #1 | Search ((breast) OR breast cancer) OR breast neoplasms | 982503 | 10:11:32 |

CINAHL

| Search number | Query | Results |
| --- | --- | --- |
| 4 | ( “breast,” OR “breast cancer,” OR “breast neoplasms,” ) AND ( “MBCT,” OR “mindfulness-based cognitive therapy,” )/lim Clinical trail | 0 |
| 3 | ( “breast,” OR “breast cancer,” OR “breast neoplasms,” ) AND ( “MBCT,” OR “mindfulness-based cognitive therapy,” ) | 13 |
| 2 | “MBCT,” OR “mindfulness-based cognitive therapy,” | 316 |
| 1 | “breast,” OR “breast cancer,” OR “breast neoplasms,” | 109812 |

PsycINFO

| **#** | Query | **Limiters/Expanders** | **Results** |
| --- | --- | --- | --- |
| S4 | breast neoplasms OR breast OR breast cancer AND mbct AND mindfulness-based cognitive therapy | Limiters - Full Text; Publication Year: 1984-2021; Published Date: -20210931; Methodology: CLINICAL TRIAL Expanders - Apply related words; Also search within the full text of the articles; Apply equivalent subjects Search modes - Find all my search terms | 180 |
| S3 | breast neoplasms OR breast OR breast cancer AND mbct AND mindfulness-based cognitive therapy | Limiters - Full Text; Publication Year: 1984-2021; Methodology: CLINICAL TRIAL Expanders - Apply related words; Also search within the full text of the articles; Apply equivalent subjects Search modes - Find all my search terms | 184 |
| S2 | breast neoplasms OR breast OR breast cancer AND mbct AND mindfulness-based cognitive therapy | Limiters - Publication Year: -2021; Methodology: CLINICAL TRIAL Expanders - Apply related words; Also search within the full text of the articles; Apply equivalent subjects Search modes - Find all my search terms | 599 |
| S1 | breast neoplasms OR breast OR breast cancer | Expanders - Apply related words; Also search within the full text of the articles; Apply equivalent subjects Search modes - Find all my search terms | 24,945 |
